# HLA Class Ia and Ib Polyreactive Anti-HLA-E IgG2a Monoclonal Antibodies (TFL-006 and TFL-007) Suppress Anti-HLA IgG Production by CD19+ B Cells and Proliferation of CD4+ T Cells While Upregulating Tregs

**DOI:** 10.1155/2017/3475926

**Published:** 2017-05-28

**Authors:** Mepur H. Ravindranath

**Affiliations:** Terasaki Foundation Laboratory, 11570 W. Olympic Blvd., Los Angeles, CA 90064, USA

## Abstract

The anti-HLA-E IgG2a mAbs, TFL-006 and TFL-007, reacted with all HLA-I antigens, similar to the therapeutic preparations of IVIg. Indeed, IVIg lost its HLA reactivity, when its HLA-E reactivity was adsorbed out. US-FDA approved IVIg to reduce antibodies in autoimmune diseases. But the mechanism underlying IVIg-mediated antibody reduction could not be ascertained due to the presence of other polyclonal antibodies. In spite of it, the cost prohibitive high or low IVIg is administered to patients waiting for donor organ and for allograft recipients for lowering antiallograft antibodies. A mAb that could mimic IVIg in lowering Abs, with defined mechanism of action, would be highly beneficial for patients. Demonstrably, the anti-HLA-E mAbs mimicked several functions of IVIg relevant to suppressing the antiallograft Abs. The mAbs suppressed activated T cells and anti-HLA antibody production by activated B cells, which were dose-wise superior to IVIg. The anti-HLA-E mAb expanded CD4+, CD25+, and Foxp^3^+ Tregs, which are known to suppress T and B cells involved in antibody production. These defined functions of the anti-HLA-E IgG2a mAbs at a level superior to IVIg encourage developing their humanized version to lower antibodies in allograft recipients, to promote graft survival, and to control autoimmune diseases.

## 1. Introduction

The humoral theory of transplantation recognizes that the high level of IgG Abs in patients waiting for donor organs and the Abs formed after transplantation are the causal factor in graft loss. Performing transplantation in patients with high levels of Abs (sensitized patients) is considered futile [1–4]. The de novo donor-specific Abs (DSA) formed against mismatched HLA molecules of different loci (HLA-A, HLA-B, HLA-C, HLA-DR, HLA-DQ, and HLA-DP) are capable of damaging the allografts [1, 5–7]. DSA may cross-react with shared epitopes on other MHC molecules [8], to augment the levels of de novo nondonor-specific Abs (NDSA) [9–12]. In addition, compatible MHC molecules (e.g., HLA-Ib antigens) overexpressed upon inflammation may elicit antibodies and contribute to the pool of NDSA. Both DSA and NDSA are capable of binding and/or aggregating on the vascular endothelial lining, attracting complement components (C1q, C4d) which form complexes that cause vascular blockage leading to minimal graft function, rejection, and graft loss [11, 12]. The allograft recipients may also develop Abs against nonclassical HLA (HLA-E, HLA-F, and HLA-G) [13] and non-MHC autoantigens (e.g., AT1R, vimentin, collagen, myosins) that may or may not be released from the allograft. Interestingly, these Abs are also correlated with loss of function of the allograft [14–18]. Several therapies are contemplated, and a few were developed to lower these Ab levels.

Ab formation depends on both T and B cells to produce Abs against allo- or autoantigens. Therefore, aggressive suppressive strategies are developed to simultaneously deplete the T and B cells, in order to suppress the development of Abs formed prior to (sensitization) or after transplantation (de novo Abs). One such aggressive immunotherapeutic strategy is induction therapy with rabbit or horse anti-human thymoglobulin, a polyreactive polyclonal mixture of nonspecific cytotoxic Abs capable of killing almost every immune cell, as documented by the list of immune cell surface antigens recognized [19].

An alternate strategy to suppress antibody formation was to transfuse polyclonal Abs purified from plasma pooled from thousands of donors, referred to as the intravenous immunoglobulin (IVIg), which either alone [20–23] or often in combination with plasmapheresis [24], or rituximab [25], a monoclonal Ab (mAb) that depletes CD20+ B cells [26]. IVIg is a complex entity consisting of polyreactive polyclonal IgG with a minor fraction of IgA Abs. Several immunosuppressive capabilities are attributed for IVIg, but its mechanism of action is far from clear, due to the polyclonality and polyreactivity of the mixture of Abs.

Most of the immunosuppressive therapies (IVIg, antithymoglobulin) involved in reducing antibody production were developed before the discovery of Tregs. It is well known now that Tregs are capable of controlling, depleting, or inhibiting CD4+ [27] and CD8+ [28, 29] T and B cells involved in antibody production [30, 31]. Tregs are also known to involve organ transplantation [32], and Tregs are found both in the recipients' lymphoid tissues posttransplantation and also at the graft sites [33].

While depleting T and B cells is important for preventing Ab formation before and after transplantation, such a therapy in combination with any therapy that induces and preserves the functionality of the tolerogenic Treg cells would be ideal and highly beneficial for allograft recipients [34], because these regulatory cells per se are potentially capable of suppressing Ab production. Although IVIg preparations were reported to suppress CD4+ T cells [35, 36], CD8+ T cells [37], and CD19+ B cells [38] and expand CD4+CD25+ Treg [39], the conflicting reports on the potential of IVIg to suppress T and B cells [40–43] cast doubt on the reliability of IVIg for depleting both T and B cells.

The polyclonality of the Abs in IVIg, or innate differences in the commercial preparations of IVIg [44], may account for the conflicting reports and doubts on its reliability. Therefore, an ideal therapeutic agent to achieve the combinatorial effect of depleting T and B cells and upregulating Treg could be a well-defined monoclonal Ab (mAb), which not only enables better understanding of its modus operandi but would also be the most cost-effective therapeutic agent than the high-dose IVIg. It could serve as an ideal and intensive therapeutic agent for desensitization and would minimize the formation of de novo DSA and NDSA to prevent Ab-mediated acute and/or chronic graft loss.

In this report, we review a series of experimental investigations made that show anti-HLA-E monoclonal IgG2a [45] binds to HLA-Ia and HLA-Ib molecules similar to IVIg preparations [46] and is simultaneously capable of depleting and arresting the functions of CD4+ T cells and CD8+ cytotoxic T cells [47], arresting anti-HLA Ab formation by CD19+/CD20− B cells [48] and upregulating the tolerogenic CD4+/CD25+/Foxp^3^+ Treg (M. Taniguichi and M. H. Ravindranath, manuscript in preparation). The objective is to offer proof that anti-HLA IgG2a mAbs are functional mimics therapeutic IVIg in suppressing T and B cells, antibody production, and upregulation of Treg.

## 2. Experimental Approach and Observations

### 2.1. Monoclonal Anti-HLA-E mAbs Mimic Polyclonal IVIg In Vitro

The pool of Abs developed in sensitized patients are removed by plasmapheresis and substituted with plasma [49] or with IVIg [24] or IVIg alone [20–23] prior to obtaining a donor organ or transplantation. Though the mechanism is far from clear, one of the immunomodulatory roles of IVIg is to lower Abs. To further suppress the Ab production in these patients, rituximab was used as a combinatorial therapy [25]. Adverse events and the costs of therapies prohibit repeated administration of some of these IVIg-combinatorial therapies.

All the therapeutic preparations of IVIg examined revealed that they contain antibodies against all alleles of HLA-A, HLA-B, HLA-Cw, HLA-F, and HLA-G loci, and it reacted with all of the classical (HLA-Ia [HLA-A:31, HLA-B:50, and HLA-Cw:16]) and nonclassical (HLA-Ib [HLA-E: 2, HLA-F:1, and HLA-G:1) molecules in a Luminex single-antigen bead assay. When the HLA-E reactivity was specifically removed from IVIg, the entire HLA-Ia reactivity of IVIg disappeared, suggesting that the HLA-Ia reactivity of IVIg could be due to the cross-reactivity of anti-HLA-E Abs in IVIg with HLA-I antigens [46]. It is hypothesized that the anti-HLA-E reactivity of IVIg could be responsible for some of the immunomodulatory activities of IVIg.

It was hypothesized that a monoclonal Ab that could mimic the immunomodulatory functions of IVIg could serve as an ideal therapeutic agent. In this regard, a unique category of mAbs came to light while examining the HLA reactivity of more than 100 mAbs generated against *β*2-microglobulin-free heavy chain of HLA-E. A set of these mAbs (such as TFL-006 and TFL-007, both IgG2a mAbs) uniquely recognized a common epitope (amino acid sequence) (^117^AYDGKDY^123^ and ^126^LNEDLRSWTA^135^) shared by almost all alleles of HLA class I loci, and it reacted with all of the classical (HLA-Ia [HLA-A:31, HLA-B:50, and HLA-Cw:16]) and nonclassical (HLA-Ib [HLA-E: 2, HLA-F:1, and HLA-G:1]) molecules in a Luminex single-antigen bead assay (Table 1) [45, 46]. Strikingly, the HLA-I polyreactivity of the anti-HLA-E^R^ mAbs is identical to the HLA-Ia reactivity of different commercial preparations of IVIg. In studying their immunomodulatory capabilities, it was noted that these polyreactive HLA-E mAbs mimic some of the functions of IVIg critical for the suppression of the production of the anti-allograft Abs [47, 48].

Therefore, we tested whether HLA-Ia and Ib-reactive anti-HLA-E mAbs mimic some of the immunoregulatory functions of IVIg. This report reviews the different immunomodulatory functions of mAbs TFL-006 and TFL-007 that mimic IVIg, and it demonstrates that the performance of the mAb in vitro is much better than IVIg.

### 2.2. mAb TFL-007 versus IVIg: Suppression of the Production of Anti-HLA Abs by B Cells In Vitro

To examine whether the anti-HLA-E IgG2a (TFL-007) suppresses the secretion of allo-HLA-II Ab by activated B^memory^ cells [48], a B cell population was separated from the peripheral blood mononuclear cells of a woman alloimmunized postpartum 23 years prior to testing, who had developed Abs directed against her husband's HLA class II antigen, after the first delivery (example number 1). In addition, we have also examined the efficacy of the mAb TFL-007 to suppress the anti-HLA-I Ab production by the immortalized B cells (hybridoma cell line, HML-416) from a woman similarly immunized postpartum by allo-HLA (example number 2).

In example number 1, the first daughter (current age 26) of the mother carried her father's nonmaternal allele, DRB1^∗^01 : 01 which may have been responsible for the presence of anti-DRB1^∗^01 : 01 IgG Ab in the mother. Fetomaternal transfer of HLA Abs and B cells is known to occur [50, 51]. Recent analysis of the maternal sera indicated that the high MFI of the anti-DRB1^∗^01 : 01 persisted in the blood 26 years after alloimmunization, suggesting that the maternal B cells producing the Abs may be long-lived B^mem^ cells. The sera also reacted with lower MFI to DRB1^∗^01 : 02, DRB1^∗^04 : 04, DRB1^∗^04 : 05, DRB1^∗^14 : 02, and DRB1^∗^04 : 01, which could be due to cross-reactivity of the primary allo-Abs anti-DRB1^∗^01 : 01 IgG, as Cai et al. [8] construed for the presence of nondonor-specific anti-HLA-II Abs in allograft recipients.

**Figure 1:**
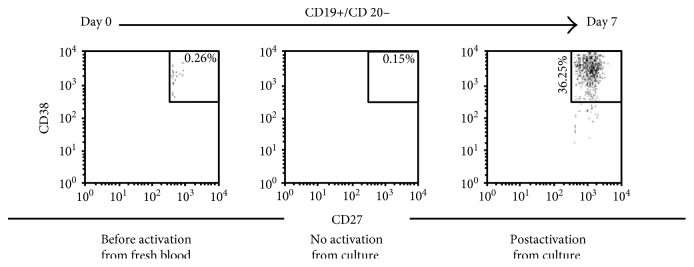
The B cells isolated from an alloimmunized woman's blood were activated in vitro by a selected battery of cytokines IL-2/IL-4/IL-6/IL-10/IL-21 (at 1/4/4/2/2 ratio) and 1 *μ*g/ml human CD40 Ab for 7 days, which resulted in an increase in plasma cells (CD19+/CD20−/CD27++/CD38++) from 0.26% on day 0 to 36.25% on day 7 [48].

The CD19+ B cells isolated from PBMC on day 0, consisted of naïve B cells (CD20+/CD27−/CD38+/−) (74.47%), B^memory^ cells (CD20+/CD27+/CD38−) (8.47%), and plasma cells (CD20−/CD27++/CD38++) (0.26%). These cells were activated in vitro with IL-2, IL-4, IL-6, IL-10, and IL-21 (at 1/4/4/2/2 ratio) and 1 *μ*g/ml human CD40 Ab for 7 days (Figure 1), which resulted in an increase in the plasma cells from 0.26% to 36.25% on day 7.

Several microplate wells contained the primary alloAb (anti-DRB1^∗^0101 IgG) with high MFI. The B cells from these wells were pooled and recultured in 4 wells on day 7 (Figure 1). On day 9, the cells were pooled and aliquoted into 3 wells and maintained without the cytokine combo or anti-CD40 mAb. These wells were exposed to medium or IVIg (1/100, 1.5 mg/ml) or mAb TFL-007s (“s” indicates the mAb purified from the supernatant) (1/100, 5 *μ*g/ml) for 72 hours. IVIg protein concentration was 300-fold higher than that of purified TFL-007s (5 *μ*g/ml) used in the treatment of B cells in culture.

**Figure 2:**
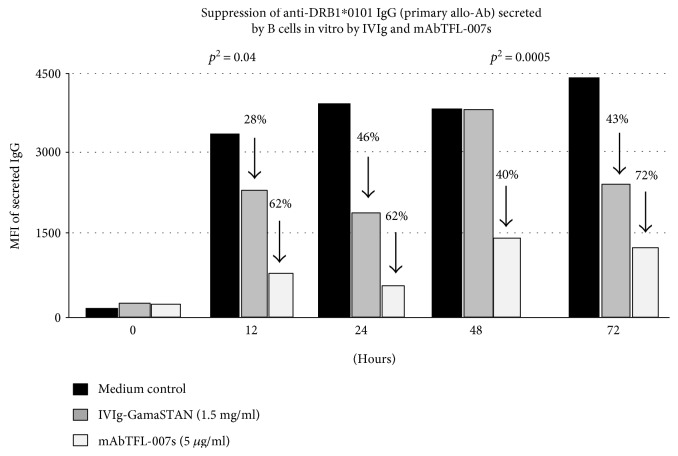
HLA molecular typing of an alloimmunized woman's family showed that the first child shared the father's HLA-II type (DRB1^∗^0101 and DQA1^∗^0101/DQB1^∗^0501). Consequently, the mother had high levels of allo-Abs (based on mean fluorescent intensity observed with Luminex single-antigen bead assay) against both the DRB and DQ alleles, even 23 years after alloimmunization, indicating the presence of long lived B^mem^ cells. The allo-Abs with affinity for husband's HLA class II [primary alleles] are designated as “primary allo-Abs.” The sera also contained “secondary allo-Abs” reacting to DRB1^∗^0102, DRB1^∗^0404, DRB1^∗^0405, DRB1^∗^1402, and DRB1^∗^0401 (possibly those cross-reactive to the primary alleles). The B cells were isolated from the fresh peripheral blood of the mother. Using Ficoll-Paque PLUS, the peripheral blood mononuclear cells (PBMC) were isolated. The B cells (resting) were isolated from the PBMC by positive selection using CD19 Pan B Dynabeads® magnetic beads. B cells were detached by DETACHaBEAD® CD19. Purified human B cells were >95% CD19+, as determined by flow cytometry analysis. Purified B cells were plated at 0.2 × 10^6^/200 *μ*l/well in a sterile 96-well, round-bottom plate. B cells were cultured in Iscove's modified Dulbecco's medium, containing HEPES, L-glutamine, and sodium pyruvate, supplemented with 10% AB human serum, 5 *μ*g/ml recombinant human (rh) insulin, 50 *μ*g/ml rh transferring, 25 μg/ml gentamicin, and 50 *μ*M 2-mercaptoethanol (2-ME). The resting B cells were activated with 25 ng/ml rh IL-2, 100 ng/ml rh IL-4, 100 ng/ml rh IL-6, 50 ng/ml rh IL-10, 50 ng/ml rh IL-21, and 1 *μ*g/ml human CD40 Ab. On day 7 of the culture, 10 *μ*l of culture supernatant from each well was analyzed for the presence of anti-HLA class II IgG allo-Abs. Cells from the wells that contained the HLA Abs were further harvested, washed three times, seeded into 4 wells, and activated as above. On days 8 and 9, the culture supernatants were tested for the secretion of allo-HLA Abs. The cells were pooled, washed (3×), and aliquoted into 3 wells: with medium alone; with GamaSTAN IVIg at 1/100 dilution, 1.5 mg/ml; and with mAb TFL-007s at 1/100 dilution containing 5 *μ*g/ml. The cells were maintained in culture without any cytokine activators or anti-CD40 Ab for an additional 3 days, and 10 *μ*l of culture supernatants from each well was analyzed for HLA allo-Abs at hours 0, 12, 24, 48, and 72. The figure shows the paired sample analysis of the triplicates at different hours. The paired sample two-tailed *t*-test was used to compare the results obtained at the stated hours with IVIg and TFL-007s against those for the control wells. The paired sample two-tailed *t*-test was carried out for IVIg and TFL-007s separately. Combined mean of the triplicate values obtained at 12 to 72 hrs for IVIg and TFL-007s was against the pooled values for the medium only of control wells (details in [48]).

**Figure 3:**
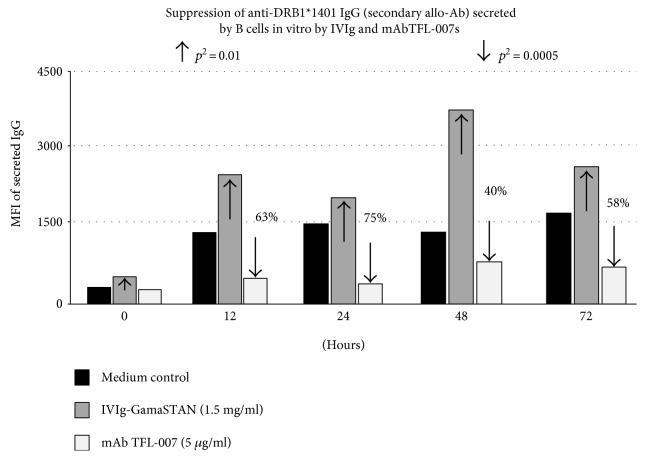
The anti-HLA-E mAb TFL-007s (conc. 5 *μ*g/ml) but not IVIg (GamaStan, conc. 1.5 mg/ml) shows inhibition of the secondary allo-Ab anti-DRB1^∗^1401 IgG. *p*^2^ (two-tailed *p* value). The details of experimental protocol are identical to those of Figure 2.

The supernatants recovered from the respective wells were screened for the HLA-allo-Abs. The levels of allo-HLA Abs are compared between those recovered from the wells that contained only the medium with those that contained IVIg or mAb TFL-007. Since IVIG reacts with most HLA molecules nonspecifically, it could be construed that any detection IgG in samples containing IVIg is interfered, causing false positivity. However, we have compared the levels of allo-HLA-Abs found in the IVIg containing wells with that of the level observed in the medium only (without IVIg).  Figure 2() reveals a critical finding for this investigation, that is, the level of allo-HLA Ab in the wells containing IVIg is significantly lower (28%, 46%, and 43% lower at 12, 24, and 72 hrs, respectively, than that of the Ab level in the well containing medium only. Indeed, IVIg (GamaSTAN) suppressed the secretion of the anti-DRB1^∗^01 : 01 IgG at different time points but only marginally (*P* < 0.04). Suppression by TFL-007s was markedly different from that of IVIg. The mAb reduced the secretion of both primary (the anti-DRB1^∗^01 : 01 IgG) and secondary Abs significantly (*P* < 0.001 to 0.0001) at levels higher than that of IVIg (Figure 2()). The percentage difference at different time intervals of secretion of the anti-DRB1^∗^01 : 01 IgG confirms the suppressive efficacy of TFL-007s. Indeed, it is mAb TFL-007s—not IVIg—that strongly suppressed the secretion of both primary and secondary allo-HLA-DRB IgG Abs (Figure 3()) which was secreted by activated normal healthy human B^mem^ cells.

**Figure 4:**
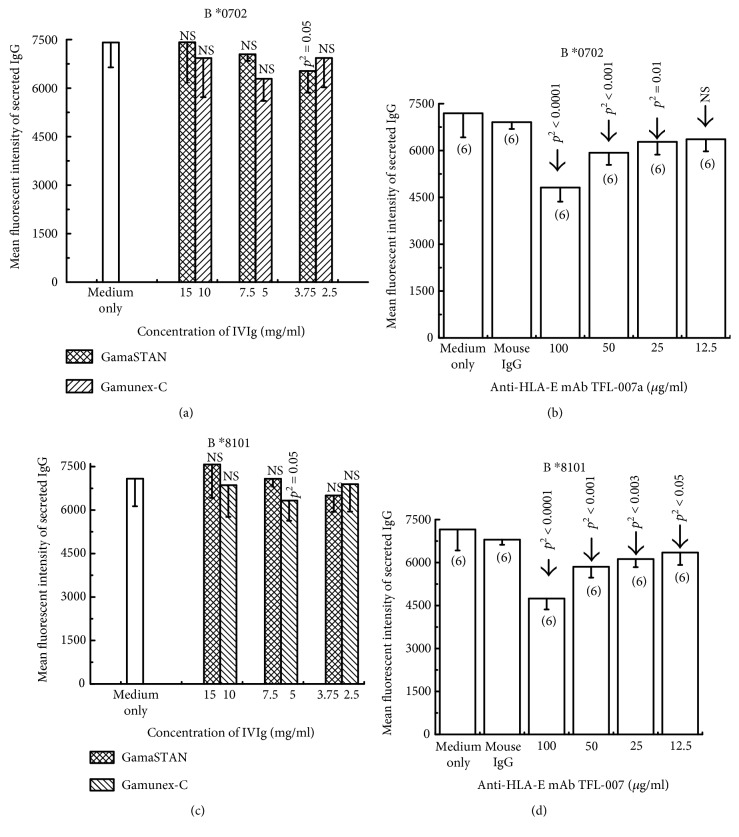
The human hybridoma cell line HML16 was generated from the resting B cells by EBV transformation; then the clone was fused with the murine, nonproducing myeloma cell line P3X63-Ag8.653 (ATCC® CRL 1580™). HML16 produced high MFI allo-Abs against B^∗^0702, B^∗^8101, B^∗^6701, and B^∗^4201 and low MFI allo-Abs against B^∗^2708, B^∗^2705, B^∗^5501, B^∗^5601, and B^∗^8201. Figures are restricted to two of allo-HLA-I Abs, namely, B^∗^0702 (a and b) and B^∗^8101 (c and d). HML16 cells (cultured in RPMI-1640 + 20% heat-inactivated fetal bovine serum + 1 mM sodium pyruvate + L-glutamine-pen-strep solution + 50 *μ*M 2-ME) were seeded at 1000/100 *μ*l/well in a Falcon 96-well flat-plate and divided into 3 treatment groups: medium control, mouse IgG control (100 and 50 *μ*g/ml), and TFL-007a. Medium control was compared with treatment by IVIg preparations (GamaSTAN and Gamunex-C; three subgroups for each), or four subgroups for TFL-007a were established for different doses except medium control. Six or more repetitions were performed with each subgroup (sample size is shown in (b) and (d)). The three subgroups of GamaSTAN-IVIg were at dilutions 1/10 (15 mg/ml), 1/20 (7.5 mg/ml), and 1/40 (3.75 mg/ml); the subgroups of Gamunex-C were at 1/10 (10 mg/ml), 1/20 (5 mg/ml), and 1/40 (2.5 mg/ml); and those of mAb TFL-007a were at 1/10 (100 *μ*g/ml), 1/20 (50 *μ*g/ml), 1/40 (25 *μ*g/ml), and 1/80 (12.5 *μ*g/ml). Twenty *μ*l of culture supernatant from each well was analyzed for allo-HLA Abs at hours 0 and 72. The anti-HLA-E mAb TFL-007a (stock 627 *μ*g/ml) at different concentrations (62.7, 32.35, 16.17, and 8.9 *μ*g/ml) but not IVIg preparations (GamaStan (15, 7.5, and 3.75 mg/ml) and Gammunex (10, 5 and 2.5 mg/ml)) inhibited the production of anti-HLA Abs against B^∗^0702 and B^∗^8102 produced by EBV-immortalized B cell line, HML-16. Mean and SD of the hexaplicate samples are presented. The paired sample two-tailed *t*-test was carried out. Two-tailed *p* values are provided in the figure [49].

It is known that the extent of IVIg-mediated apoptosis of resting and activated human B cells was significantly lower than that observed with B cell hybridomas [42]. Therefore, we examined the ability of IVIg and TFL-007a (“a” indicates ascites purified mAb) to suppress the secretion of anti-HLA-I Abs secreted by the hybridoma (HML16) (example 2). HML-16 cell line produced anti-HLA-I Abs with high MFI against B^∗^0702 > B^∗^8101 > B^∗^4201 > B^∗^6701. Two different preparations of IVIg (GamaSTAN and Gamunex) were used with a media control. A high-dose IVIg, used in transplant patients, was used to suppress the Ab production by the hybridoma. IVIg-GamaSTAN (at dilutions 1/10, 1/20, and 1/40 with dosages 15 mg/ml, 7.5 mg/ml, and 3.75 mg/ml, respectively) and IVIg-Gamunex (at dilutions 1/10, 1/20, and 1/40, with dosages 10 mg/ml, 5 mg/ml, and 2.5 mg/ml, respectively) were used. Neither of the IVIgs suppressed the secretion of allo-HLA-B IgG by the hybridoma cells (Figures 4(a) and 4(c)). In striking contrast, the mAb TFL-007a significantly suppressed the secretion of both anti-HLA-B^∗^0702 and anti-B^∗^8101 IgG Abs (Figure 4(b) and 4(d)). Most importantly, TFL-007a showed dosimetric suppression of allo-HLA-I Abs. Indeed, anti-HLA-E mAb TFL-007a, in marked contrast to IVIg preparations, significantly suppressed the secretion of both allo-HLA-B Abs.

The comparison between the potential of therapeutic IVIg and monoclonal anti-HLA-E IgG2a (TFL-007) in suppressing the production of IgG Abs formed against (1) paternal-specific HLA-II antigen and cross-reactive HLA-II antigens by B cells (CD19+/CD20−/CD27+/CD38+) in a mother (example number 1) and (2) paternal HLA class I antigens by EBV immortalized B cells obtained from a mother (example number 2) confirmed that the immunosuppressive potential of anti-HLA-E IgG2a mAb is superior to IVIg. This exemplifies a cost-effective immunosuppressive therapeutic strategy by utilizing the mAb for desensitization of patients prior to transplantation as well as to suppress effectively both donor-specific and nondonor-specific HLA-I and HLA-II Abs.

### 2.3. TFL-007 or TFL-006 versus IVIg: Suppression Activated CD4+ and CD8+ T Cells In Vitro

The effects of different concentrations of IVIG on phytohemagglutinin (PHA) activated T lymphocytes were examined in vitro [52], and it was observed that IVIg controls the T lymphocyte activation, possibly by binding to the specific Fc-receptor expressed on the surface of activated T cells. Subsequent reports documented that IVIg suppresses cytokine-activated T lymphocytes [35], by apoptosis [38], by arresting the production of cytokine involved in the activation of T cells [53], and by suppressing proliferation of human (auto) antigen-specific T cells without inducing apoptosis [41]. The exact mechanism of suppression of T cell functions could not be defined unequivocally, as IVIg contains all of the subclasses of IgG Abs (Octogam: IgG1 65%, IgG2 30%, IgG3 3%, and IgG4 3%), their F(ab)^2^ fragments and varying concentrations of IgA and lower amounts of IgM along with T-helper type 2 (Th2) cytokines and cytokine antagonists in different preparations of IVIg [54]. Furthermore, IVIg is prepared by purifying IgG from plasma, pooled from 10,000 to 60,000 donors, that contains several undefined antigen-specific and polyreactive Abs. In spite of the ambiguity, IVIg, in pre- and posttransplant patients, is considered to reduce T cell activation and proliferation (blastogenesis) in allograft recipient, and to suppress the production of anti-allograft Abs, biopsy-proven T cell-mediated allograft rejection and T-lymphoproliferative disorders developed posttransplantation [55, 56]. Since we observed that the performance of anti-HLA-E IgG2a mAb (TFL-007) was superior to IVIg in mimicking the suppression of anti-HLA body production by B^memory^ cells, we have also examined whether mAb TFL-007 and another closely related but similar IgG2a mAb TFL-006 are capable of suppressing blastogenesis and proliferation of CD4+ and CD8+ T lymphocytes [47], with appropriate controls.

For in vitro experimental purposes, T lymphocytes were recovered from the peripheral blood of mononuclear cells (PBMCs) of healthy human donors and isolated using Ficoll™-Hypaque (GE Healthcare BioSciences Corp., Piscataway, NJ, USA) and for isolating the lymphocytes LymphoKwick® (One Lambda) was used. The isolated lymphocytes were separated into two batches, one activated with phytohaemagglutinin (PHA) at a final concentration of 2·25 *μ*l/ml and the other not activated (PHA-negative control). The CD4+ or CD8+ lymphoblasts were identified by the size (forward scatter) and by the granularity (side-scatter), using Flow cytometry. Blastogenesis of activated T cells and proliferation was monitored with CFSE, a cell-permeable dye [57]. IVIg- or mAb-mediated suppression of proliferation was recorded for cessation of mitosis, as measured by the successive twofold reductions in the CFS intensity after 72 h of treatment.

**Figure 5:**
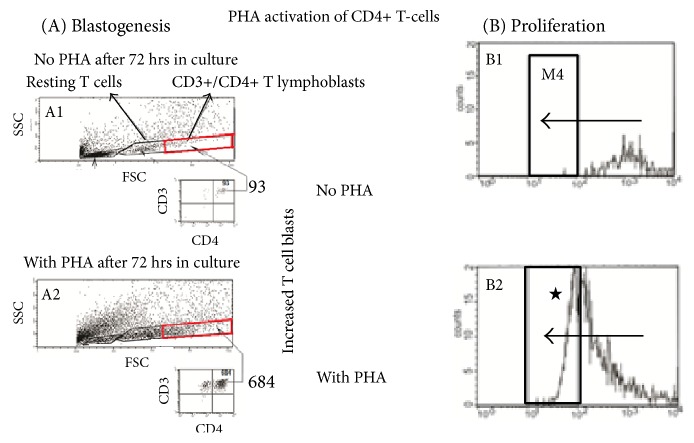
Phytohemagglutin (PHA) mediated activation of CD3+/CD4+ T lymphocytes. PHA induces blastogenesis and proliferation of the T cells. Experiments were done in triplicate (see [47]). Blastogenesis of was determined by counting the lymphoblasts, after culturing purified lymphocytes with or (as control) without PHA for 72 h. Proliferation was monitored by labelling the purified lymphocytes with the intracellular fluorescent dye carboxyfluorescein succinimidyl ester (CFSE). After 72 h, the labelling of the cells was measured: PHA-treated T cells undergo four to six divisions. Using flow cytometry, the mitotic activity is measured by the successive twofold reductions in fluorescent intensity of the T cells placed in culture for 72 h. Experiments were done in triplicate [48]. In the no PHA box, the number of cells in the M4 column is highly negligible. The box “with PHA” shows increase in the cell numbers in M4 column (shown with ^★^).

**Figure 6:**
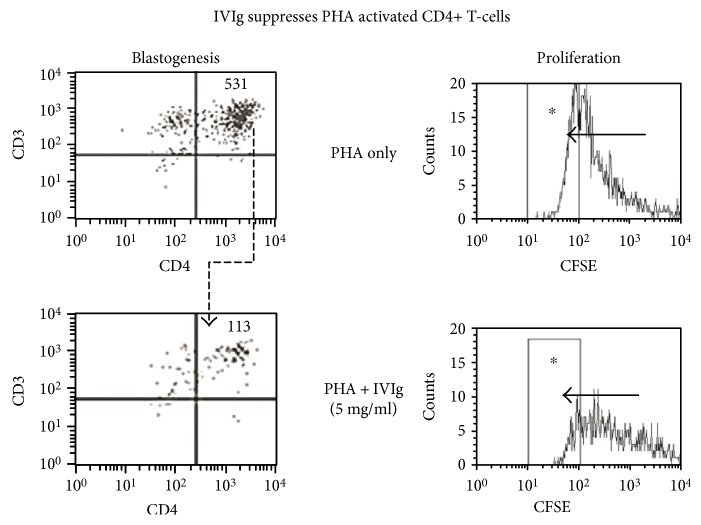
IVIg (Glob EX, VHB Life Sciences Limited, Mumbai, India) at a concentration of 5 mg/ml (at dilution 1/10) suppressed blastogenesis and proliferation of the PHA activated CD3+/CD4+ T lymphocytes. Experiments were done in triplicate (see [47]). When the cells divide, the CFSE is passed on to the progeny, as indicated in the upper box by the migration of staining from right to left (marked as ∗ in a rectangular area) at every sequential mitosis, with the number of cell divisions (mitosis 4 (M4)) determining the distance moved. Addition of IVIg (5 mg/ml) to wells with PHA suppressed the proliferation as indicated in the lower box within the rectangular area (marked as ∗). For details, see [47].

IVIg or mAbs were added to the cells in culture by the addition of PHA over the course of two hours (the total volume was adjusted to 200 *μ*l), based on a previous report that evaluated the effects of time differences in the addition of IVIg and other toxins after adding PHA [53]. After PHA treatment, the CD4+ T lymphoblast cell density increased five- to sixfold over the PHA-negative control (Figure 5(a)). Similarly, PHA-activated T lymphocytes proliferated dramatically (Figure 5(b)). On the other hand, IVIg strikingly suppressed the PHA-activated blastogenesis of lymphocytes (Figure 6). The density of the PHA-activated T cells decreased significantly in a dosimetric fashion, indicating that IVIg potentially suppresses PHA activated T-lymphoblasts. Similarly, CFSE profiles elucidated the cessation of activated T cell proliferation by IVIg (Figure 6).

**Figure 7:**
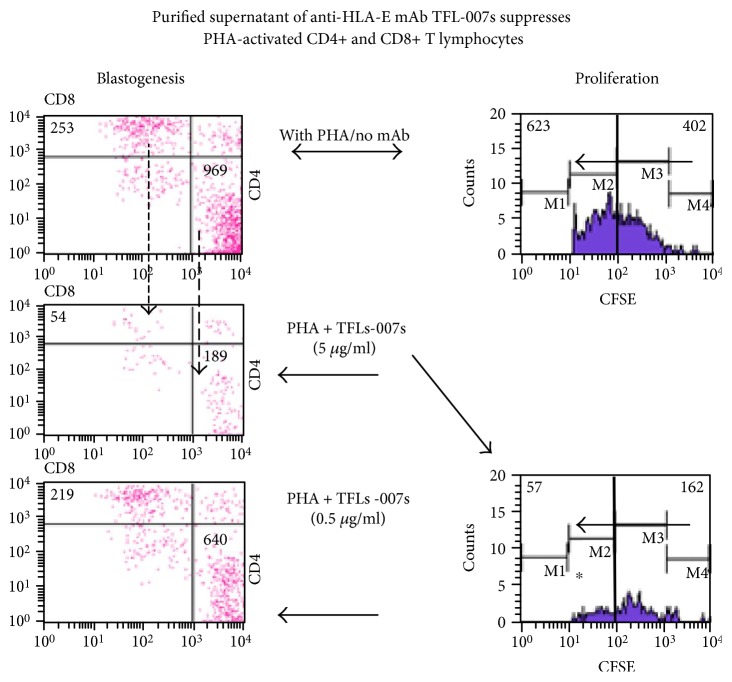
Dose-dependent inhibition of PHA-activated CD4+/CD8+ T cells in vitro with anti-HLA-E mAbs, TFL-007s (“s” for culture supernatants). The T cells were stained with PE-labeled anti-CD4 mAbs (*x*-axis) and PerCP-labeled anti-CD8 mAbs (*y*-axis). The profile is divided into three groups (only group 3 is represented in the figure; for details, see [47]) based on staining and size of cells to illustrate the differences in the CD4+ and CD8+ T cell populations and number of events. Group 1 comprises resting CD4+ and CD8+ lymphocytes, group 2 resting CD4+ and CD8+ lymphocytes, and group 3 CD4+ and CD8+ lymphoblasts. Flow cytometric profiles of PHA-treated CD4+ T cells (lower right of the boxes) and CD8+ T cells (upper left) from a normal non-alloimmunized donor (R) after treatment with mAb TFL-007s. The top row (treated only with PHA) shows the number of CD4+ T cells and the CD8+ T cells. The middle row (PHA and mAb TFL-007s at 1/10 dilution or 5 *μ*g/ml) shows the number of both CD4+ (*p*^2^ < 0.001) and CD8+ (*p*^2^ < 0.002) T cells have decreased significantly. In comparison, the bottom row with the same treatment, but at 1/100 dilution (0.5 *μ*g/ml), showed a dose-dependent decrease in the number of PHA-activated CD4+ (*p*^2^ < 0.004) and CD8+ (not significant) T lymphocytes. Each block of figure represents one of the triplicate analyses (for further details, see [47]).

**Figure 8:**
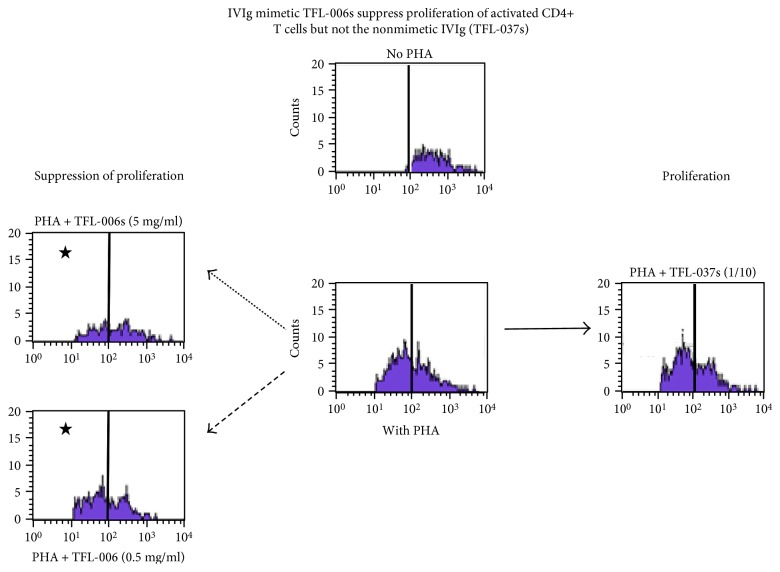
Suppression of proliferation of the PHA-activated CD4+ T cells by purified culture supernatant of anti-HLA-E mAb TFL-006s (at 5 *μ*g/ml and at 0.5 *μ*g/ml) but not by the control mAb TFL-037 (at 5 *μ*g/ml) (for details, see [47]).

The ability of the novel anti-HLA-E^R^ IgG2a mAb (TFL-007) to suppress the blastogenesis and proliferation of CD4+ and CD8+ T lymphocytes (Figure 7) is strikingly parallel to another anti-HLA-E IgG2a mAb (TFL-006) (Figure 8). At the same time, control antibodies such as anti-HLA-I reactive Abs and anti-HLA-E IgG2b mAb (TFL-037) are less reactive to HLA-I and nonreactive to HLA-F and HLA-G, and a HLA-E monospecific mAb, TFL-033, failed to suppress either blastogenesis or proliferation of PHA-activated T cells (Figure 8, Table 2).

Furthermore, in comparing the dose-dependent suppression of blastogenesis and proliferation of activated T cells of IVIg versus the mAb TFL-006 (both supernatant and that purified from the ascites), while bearing in mind the concentration of the agents used, one may witness the superiority of TFL mAbs over IVIg (Table 3), For additional details on lesser performance by IVIg, see the detailed figures in [47].

### 2.4. TFL-007 and TFL-006 versus IVIg: Upregulation of CD4+ CD25+ Foxp^3^ Tregs In Vitro

**Figure 9:**
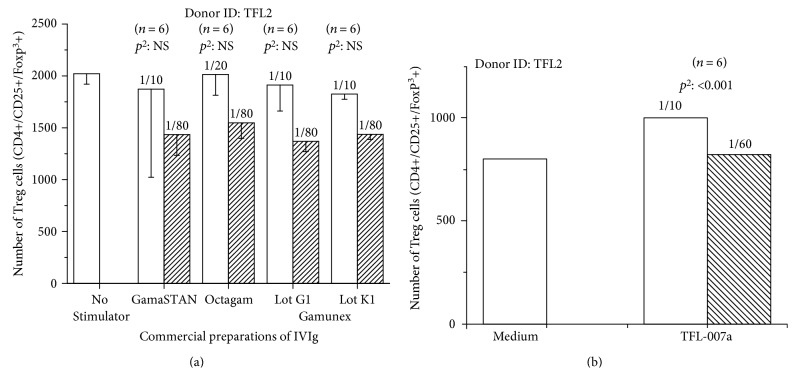
Effects of different commercial preparations of IVIg which include GamaSTAN™ S/D (15–18 gm%, lot 26NHCVI; Telacris Biotherapeutics Inc) at dilutions 1/10 (conc. 15 mg/ml) and 1/80 (conc. 1.2 mg/ml), Octagam® (6 gm%, lot A913A8431; Octapharma Pharmazeutika) at dilutions 1/20 (conc. 3 mg/ml) and 1/80 (conc. 0.75 mg/ml), and Gamunex®-C (10 gm%, lots 26NKLG1 and 26NKLK1, Telacris) at dilutions 1/10 (conc. 10 mg/ml) and 1/80 (conc. 8 mg/ml) (a) and mAb TFL-007a (at dilution 1/10, conc. 62.7 *μ*g/ml; 1/80, conc. 7.84 *μ*g/ml) (b) on PHA-untreated cells were compared with the effect of medium alone on the proliferation of Treg cells, defined as CD4+/CD25+/Foxp^3^+. Note that IVIg preparations used in this study failed to upregulate Tregs in contrast to TFL-007a which significantly upregulates Treg cells. *p*^2^ (two-tailed *p* value) (M. Taniguchi and M. H. Ravindranath, manuscript in preparation).

The CD4+ CD25+ Foxp^3^+ regulatory T cells (Tregs) are not only found in circulation [30] but also present at the site of the allograft [33]. They suppress Ab production by downregulating B memory and plasma cells [32] and depleting CD4+ [27] and CD8+ [28, 29] T cells that play a major role in graft rejection [34]. IVIg is known to upregulate Tregs [39]. The ability of anti-HLA-mAbs TFL-006 and TFL-007 to induce proliferation of CD4+CD25+Foxp^3^+ Tregs obtained from normal and healthy donors was assessed (M. Taniguchi and M. H. Ravindranath, manuscript in preparation). In this study, we have compared the impact of IVIg with polyreactive anti-HLA-E mAb TFL-007 on untreated and PHA-treated isolated fractions of CD3+/CD4+ human T lymphocytes. A variety of cell surface markers, which include CD4, CD25 (IL-2R*α*), CD45RA, and Foxp^3^, were monitored using their respective monoclonal Abs. To illustrate this proof of principle, the effect of different commercial preparations of IVIg (Figure 9(a)) and anti-HLA-E mAb TFL-007 (Figure 9(b)) were studied (in triplicate) on the untreated T-regulatory cells (CD4+/CD25+/Foxp^3^+) obtained from a healthy volunteer (TFL2). The mAb purified from ascites was used throughout. Figures 9(a) and 9(b) illustrate that the different commercial preparations of therapeutic IVIg at two different dilutions (1/10 and 1/80) failed to upregulate the Tregs, while mAb TFL-007a showed a significant increase in the number of cells compared to the controls. Further elaboration of the experiments will be available in a manuscript to be submitted shortly.

## 3. Discussion

### 3.1. The Enigma of IVIg: Problems and Solutions

#### 3.1.1. Clinical Applications of IVIg

IVIg has been used for the treatment of several autoimmune (*n* = 6) and hematological diseases (*n* = 18), neuropathies (*n* = 14), cardiomyopathies, nephropathies (*n* = 4) including acute renal failure (ARF), congenital heart block, and eye and ear diseases, asthma and cystic fibrosis, recurrent pregnancy loss, diabetes mellitus, burns, chronic fatigue syndrome, and other syndromes, such as Rasmussen, Reiter, and Vogt-Koyanagi-Harada syndromes, and several viral infections including HIV [55]. IVIg is administered at a high dose (generally 1-2 gms per kg body weight) to decrease the severity of the immune response in patients with autoimmune diseases.

In spite of the extensive use of IVIg, the US Food and Drug Administration (FDA) has cautiously approved the use of IVIg for (1) Kawasaki disease, (2) immune-mediated thrombocytopenia, (3) primary immunodeficiencies, (4) hematopoietic stem cell transplantation (for those older than 20 yrs), (5) chronic B cell lymphocytic leukemia, and (6) pediatric HIV type 1 infection. Since 2004, the Canadian Blood Services and Canada's National Advisory committee on blood and blood products initiated and developed guidelines for the use of IVIg for sensitized patients who undergone solid organ transplantation [56]. In 2004, the US Medicare approved the Cedars-Sinai Hospital (Los Angeles, CA) IVIg protocol to minimize HLA Abs in patients waiting for donor kidneys so that such recipients could accept a living or deceased donor kidney.

#### 3.1.2. Major Concerns about the Use of IVIg for Transplant Patients

The main concern for the cautious approval by FDA is mainly due to serious adverse side effects that occurred after infusion IVIg, such as anaphylactic shock, renal insufficiency, Steven-Johnson syndrome, aseptic meningitis, thromboembolic events, thrombosis, cytopenia, hemolysis, stroke, seizure, loss of consciousness, acute respiratory distress syndrome, pulmonary edema, acute bronchospasm, transfusion-associated lung injury, aseptic meningitis, delayed hemolytic reaction, acute myocardial infarction, and even acute renal failure [55, 56].

Thrombotic complications associated with the use of IVIg have been reported in twenty-nine cases including acute myocardial infarction, cerebral infarction, pulmonary embolism, deep venous thrombosis, hepatic veno-occlusive disease, and spinal cord ischemia. For renal toxicity alone, there are 32 reports published involving 78 patients in whom toxicity developed in association with IVIG treatment [58]. From June 1985 to November 1996, the FDA received 120 reports worldwide, 88 from the United States [59]. Between 1992 and 1998, 49 cases of ARF were reported to the French Regional Pharmacovigilance Center with marked creatinine increase after the initiation of IVIg therapy [60]. Transient renal failure mainly occurs when using sucrose-containing IVIg, owing to osmotic injury [61]. Specific adverse side effects were attributed to differences in osmolality, pH, and sugar and sodium content of the IVIg products.

Some of the adverse effects are attributed to HLA-II Abs in IVIg. There are several reports of occurrence of transfusion-related acute lung injury (TRALI) with one death after IVIG administration [62–67]. The mechanism underlying induction of TRALI by IVIg has been an enigma. Anti-HLA-II IgG observed in patients after plasma transfusion is implicated in TRALI [68]. The mechanism underlying the lung injury by IVIg has been clarified in humans [68]. The anti-HLA-II IgG binding to monocytes in patients with TRALI may induce the activation of neutrophils that may penetrate the endothelium of lungs, causing destruction of the endothelial cells [69]. Since the presence of HLA-II Abs in alloimmunized females led to the prevention of using blood from females for transfusion, avoidance has become routine as a preventive measure against TRALI in several countries [62, 70]. It was reported that “this policy did indeed significantly reduce the incidence of TRALI both in large-scale surveillance studies and haemovigilance reports” [70].

All these observational studies warn against using IVIg, particularly high-dose IVIg, for patients waiting for donor organs and such use of IVIg should be preceded by titer tests of HLA Abs, because there is a distinct possibility that one of the active agents in IVIg is actually the anti-HLA Ab itself. Consequently, a balancing of the danger of TRALI must be carefully considered, and the effective action of the HLA Ab must be monitored when using IVIg. Therefore, failure of functional recovery by transplanted organs or their hyperacute rejection could be a consequence of IVIg used for desensitization.

The lead clinical investigators [20–25] used high-dose IVIg for desensitization therapy in patients waiting for organ donors or for lowering antiallograft Abs posttransplantation, changed from monotherapy of IVIg to combinatorial therapy with IVIg. There could be many reported and unreported reasons for the shift, but it could also be due to the increase of the desensitization efficiency, reduction of desensitization period (3 months to 1 month), and the cost. The combinational therapies involving IVIg is further improved for graft survival by expanding the use of IVIgs with anti-IL-6R Ab tocilizumab [71] and also with an IgG-degrading bacterial enzyme IdeS (IgG endopeptidase) [72].

In spite of these reports, others reported that IVIg failed to lower the mean percentage of pretransplant HLA Abs observed before IVIg infusion (85% before, 80% after IVIg administration) [73]. Paradoxically, in another patient cohort, an increase in the level of anti-HLA-I Abs was observed after IVIg treatment in 27% of the patients [74]. The investigators further validated that the calculated PRA did not reveal any significant changes in response to IVIg therapy. Most importantly, Marfo et al. [75] showed that IVIG together with rituximab treatment failed to reduce PRA levels or the mean fluorescent intensity of HLA Abs as measured in Luminex single-antigen bead assays in the patients.

#### 3.1.3. The Enigma of the Immunomodulatory Effects of IVIg

IVIg contains polyreactive natural Abs, which include IgG against endogenous and exogenous Abs, immunomodulating peptides, all the blood group antigens, and various cytokines. While few of the immunoregulatory mechanisms of action of IVIg have been proven, many proposed mechanisms still remain an enigma, due to polyreactivity, polyclonality, and diversity in the preparations of IVIg. Sapir and Shoenfeld [76] enlist these mechanisms as follows: (a) Fc-receptor blockade; (b) neutralization of pathogenic autoAbs via idiotypic and anti-idiotypic Abs; (c) effects on the Fas apoptotic pathway via agonistic and antagonistic anti-Fas autoAbs; (d) regulation of complement components; (e) modulation of cytokine secretion; (f) hindrance of natural-killer cell activity; (g) inhibition of matrix metalloproteinase-9; (h) suppression of NFkB activation and IkB degradation; (i) G1 cell cycle arrest; (j) prevention of tumor growth; (k) decrease in leukocyte recruitment; (l) attenuation of T cell stimulation; (m) effects on Ab kinetics; and (n) effects on dendritic cells. It is believed that various mechanisms of IVIg cooperate in a synergistic way.

We have reported earlier [47] that both blastogenesis and proliferation of activated T cells induce transitory expression of more than ten molecules enlisted earlier [47], which include IL-2R, Fc receptors for IgG, receptors of insulin, insulin-like growth factor, *α*fetoprotein, and transferin receptors, MICA, HLA-II, and *β*2-microglobulin-free heavy chain (HC) of HLA-I. It is often discussed that IVIg binds to the Fc-receptor for immunoregulation. Specifically, the inhibition of blastogenesis and proliferation of activated T and B cells by IVIg is attributed to Fc receptors for IgG (Fc*γ*RI (CD23), Fc*γ*RII (CD32), Fc*γ*RIII (CD16), and Fc*ε*R1 (CD64)) expressed on the immune cells upon activation [77]. It is far from clear as to how all of the four subclasses of IgG present in IVIg can simultaneously block the following Fc-receptors: Fc*γ*RI CD23, Fc*γ*RII/CD32, Fc*γ*RIII/CD16, and Fc*ε*R1/CD64, upregulated upon the activation of T and B cells. Paradoxically, another report [78] documented clearly that the inhibition of Ab production by B cells in vitro is brought about by F(ab')2 fragments of Abs but not by the Fc portion. The primary enigma revolves around the mechanism of action of IVIg, namely, its polyclonality that prevents specific recognition of the modus operandi of IVIg in lowering or depleting the Ab-producing B cells or the antigen-presenting T cells or the upregulation of Tregs.

Furthermore, it is highly paradoxical that IVIg is used to lower HLA Abs despite different formulations of IVIg per se which contains IgG Abs reacting to HLA-I (HLA-A, HLA-B, HLA-Cw, HLA-E, HLA-F, and HLA-G) [46] and HLA-II (DRB, DQA/DQB, and DPA/DPB) [79, 80]. Although most of the manufacturers have made efforts to prepare IVIg devoid of sucrose, which was considered as a major cause of adverse reactions, the aforementioned findings emphasize that the pharmaceutical manufacturers should document the level of HLA-II Abs as well as the levels of the soluble forms of HLA-Ia, HLA-Ib, and HLA-II antigens present in their therapeutic preparations of IVIg, particularly when they recommend IVIg for lowering HLA Abs pre- and posttransplantation.

In spite of these adverse effects and conflicting reports, the demand for therapeutic IVIg has steadily increased each year since 1992, which has resulted in product shortages and increased market prices [81]. However, there are intrinsic limitations with respect to the conventional production of therapeutic IVIgs. The quantities of human plasma that can be collected from donors are limited. The only cost-effective, evidence-based immunoreactive and immunomodulatory strategy is to substitute IVIg with IVIg mimetics. The development of such IVIg substitutes or mimetics would stabilize and even reduce the use of donor plasma-derived IVIg, thereby securing such IVIg supplies for the most restricted and life-threatening immunodeficiency diseases.

### 3.2. Monoclonal Anti-HLA-E IgG2 as IVIg Mimetics

**Figure 10:**
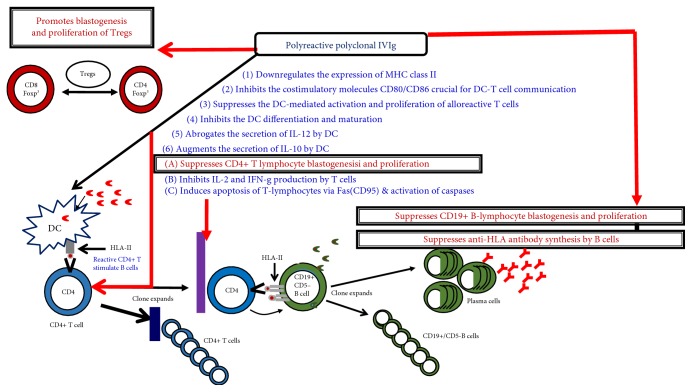
Different immunoregulatory roles attributed for the polyreactive polyclonal IVIg and proven functions of anti-HLA-E IgG2a monoclonal Abs. We have compared three functions of IVIg with that of anti-HLA-E IgG2a monoclonal Abs. We have monitored (1) suppression of CD19+ B lymphocyte blastogenesis, proliferation, and anti-HLA-I and anti-HLA-II IgG Abs, (2) suppression of blastogenesis and proliferation of CD4+ as well as CD8+ T lymphocytes, and (3) upregulation of blastogenesis and proliferation of Tregs. Our results indicate that the performance of anti-HLA-E mAbs TFL-006 and TFL-007 (the functions are recorded in red) is far superior to IVIg in achieving these functions [47, 48].

A reasonable alternative could be a monoclonal Ab that dosimetrically mimics one or more of the immunoregulatory mechanisms of IVIg (Figure 10) underlying suppression of antibody production but at the same time superior in its performance. This study evaluated and compared the immunomodulatory efficacy of HLA-I polyreactive anti-HLA-E monoclonal Abs TFL-007a and TFL-006a with that of a similarly polyreactive mixture of polyclonal Abs pooled from thousands of donor blood and purified as IVIg. Indeed, anti-HLA-E mAbs TFL-006 and TFL-007 are capable of performing three of the major immunoregulatory functions far more than IVIg. The three functions defined in this report are as follows:

1. Suppression of CD19+ B lymphocyte blastogenesis, proliferation, and suppression of anti-HLA-I and anti-HLA-II IgG Abs
2. Suppression of blastogenesis and proliferation of CD4+ as well as CD8+ T lymphocytes
3. Expansion of CD4+, CD25+, and Foxp^3^+ Tregs.

It is this report and earlier publications that document the experimental details that show TFL-006 and TFL-007, the IgG2 mAbs [45–48], bind to the surface antigens (HLA-Ia and HLA-Ib) on CD4+ T cells, CD8+ T cells, and CD19+ B cells to bring about the observed effects. The mAbs may bind to CD4+ T cells which are also positive for CD25 and Foxp^3^ and perform a function quite opposite to what it does, when it binds to other activated CD4+ T cells, which are CD25 negative and Foxp^3^ negative. The fundamental question is how does the mAbs distinguish CD4+/CD25− T cells from CD4+/CD25+/FoxP^3^ cells to perform the opposite function. Can there be two different receptors or mechanisms of action for the mAbs?

We have discussed earlier as to how the mAbs TFL-006/TFL-007 may bind to HLA-E present on activated T and B cells [47, 48]. Of various molecules upregulated upon activation of T and B cells [47], two are probable targets for mAbs TFL-006 and TFL-007. They are

1. expression of Fc*γ*Receptor IIa (CD32) [82],
2. overexpression of heavy chain (HC) HLA-I molecules without *β*2-microglobulin called “open conformers” [83, 84].

The mAbs TFL-006 and TFL-007 are IgG2a, and at present, we are not certain whether receptors for IgG2a, namely, Fc*γ*RIIa (CD32), are specifically upregulated in the human T and B cells we have examined. It is known that IgG2a binds specifically to Fc*γ*RIIa (histidine at position 131), whereas IgG2a may bind to Fc*γ*RIIb in conjunction with Fc*γ*RI [85].

Alternately, the mAbs TFL-006 and TFL-007 may recognize a shared epitope on all HLA-I molecules. A typical structure of the intact HLA-I molecule consists of HC with *β*2-microglobulin. The shared epitope is masked by *β*2-microglobulin on HC of HLA-Ia and HLA-Ib and hence considered cryptic. Most interestingly, the HLA-I molecules are expressed uniquely as *β*2-microglobulin-free HLA class I *α*-chains on activated T and B cells [85–88], and furthermore, it is already known that *β*2-microglobulin-free HLA class I HC on activated T cells can serve as ligands for leukocyte receptors [89]. The HC contains three helical structures called *α*1, *α*2, and *α*3 helices. The *α*1 domain is most susceptible to structural changes, as the *α*2- and *α*3 domains contain disulfide bonds. Therefore, one may consider the *α*1 domain as a wobbling helical domain or simply a wobbler. The wobbling of the *α*1 domain may enable binding of the mAbs TFL-006/TFL-007 to their epitope on HLA-E that is shared with all other HLA-Ia molecules. The mAbs TFL-006/TFL-007 were raised by immunizing the folded *β*2m-free HLA-E^R107^. The epitope of TFL-006 is shared by HLA-A/HLA-B/HLA-Cw/HLA-E/HLA-F and HLA-G, and the location of the sequence (shown in figures [90]) is cryptic in the *β*2-microglobulin-associated HC since *β*2-microglobulin masks the epitope. The epitope of the mAbs TFL-006 and TFL-007 is identified by inhibiting their binding to HLA-E-coated beads by the most common and accessible shared peptide sequences located on the *α*2 domain of HC of HLA-E which include ^117^AYDGKDY^123^ and ^126^LNEDLRSWTA^135^ [47]. The ability of TFL-006 and TFL-007 to bind to regular HLA beads or acid-treated beads but not to iBeads, which are coated with *β*2-microglobulin-associated HCs of HLA-Ia, confirms that the epitope affinity of the mAbs TFL-006 and TFL-007 is *β*2-microglobulin-free HC of HLA-Ia. In view of its unique property of TFL-006 in recognizing *β*2-microglobulin-free HC of HLA-Ia, the works of Jucaud et al. consider it as a potential diagnostic tool to distinguish *β*2-microglobulin-associated HC of HLA-Ia from *β*2-microglobulin-free HC of HLA-Ia coated on Luminex single-antigen beads (One Lamda Inc/Thermofisher Inc) extensively used in monitoring HLA Abs in patients waiting for donor organs and those who underwent transplantation [91, 92]. Furthermore, the suppression of blastogenesis and proliferation by mAbs TFL-006 and TFL-007 but not by mAbs TFL-033 (IgG1) and TFL-037 (IgG2b), which do not bind to shared peptide sequences of the open conformers of HLA-I, further confirmed that the mAbs TFL-006 and TFL-007 bind to the epitopes exposed on the open conformers of HLA-E as well as other HLA-I molecules.

Furthermore, the above inferences are well supported by the fact that the HLA-I open conformers have extended cytoplasmic tails with the exposure of on otherwise cryptic tyrosyl residue at position 320 [93, 94] and serine at position 335, the sites for phosphorylation [95, 96]. Such elongation of the coiled cytoplasmic tail as in intact *β*2-microglobulin-associated HC may facilitate the wobbling of the *α*1 domain of HC and expose the HLA-I common and shared epitope for binding by TFL-006 and TFL-007. Therefore, it appears that the binding of TFL-006 or TFL-007 to the shared epitopes on the *α*1 domain on the open conformer may be involved in tyrosine and/or serine phosphorylation, which may lead to signal transduction to arrest proliferation and blastogenesis of T and B cells. At the same time, it may be involved in upregulation, depending on whether Tyrosine^320^ or Serine^335^ is involved. The TFL-006-/TFL-007-mediated phosphorylation of the cytoplasmic tails can induce dephosophorylation of T and B cells by activating phosphates, leading to the arrest of transcription factors and synthesis of proteins associated with proliferation. The reverse phenomenon may occur with CD4+/CD25+/Foxp^3^+ Tregs. This may involve any or all HLA-I molecules or specifically HLA-E expressed on these activated T cells [97]. However, at present, simultaneous binding of mAb TFL-006 or TFL-007 to Fc*γ*RIIa (CD32) and *β*2-microglobulin-free HC of HLA-I (open conformer) or binding the F(ab') of mAb to the open conformers after Fc of mAb binding to Fc*γ*RIIa (CD32) cannot be ruled out. Such differential binding may account for the multifunctional capabilities of mAbs TFL-006 and TFL-007.

## 4. Conclusion

The observations reviewed in this report conclusively document that the HLA-I polyreactive anti-HLA-E IgG2a monoclonal Abs (TFL-006 and TFL-007) mimic not only the HLA-I reactivity of IVIg but also some of the critical functions such as (1) suppression of blastogenesis and proliferation of CD4+ T cells and CD8+ T cells, (2) effective inhibition of the production of anti-HLA-I and HLA-II Abs (such as “donor specific abs” formed against HLA-mismatched allografts and allo-HLA antibodies in transplant patients developed while waiting for a donor organ), and at the same time, (3) the upregulation of Tregs, which by themselves are capable of suppressing CD4+ T cells and CD8+ T cells and antibody production by B cells.  Table 4 compares the unique features of the mAbs TFL-006 and TFL-007 with that of IVIg. The data presented in this review, as well as those presented earlier [45–48], indicate that the performance of the mAbs are indeed superior to IVIg, particularly in the following aspects:

1. Unlike IVIg, the anti-HLA-E IgG2a mAb TFL-007 prevented anti-HLA Ab production by activated B cells. It is highly possible that TFL-007 can also suppress other antiallograft Abs produced by B cells.
2. Both anti-HLA-E IgG2a mAbs TFL-006 and TFL-007 are capable of suppressing antispecific activated T cells but have a dosimetrically superior performance over suppression by IVIg. Such a therapeutic mAb is invaluable for preventing autoimmune diseases and lowering Abs in allograft recipients. A version of the mAb adapted for human use will fulfill the goal that is targeted by this review.
3. Both anti-HLA-E IgG2a mAbs TFL-006 and TFL-007 have the unique potential to upregulate CD4+, CD25+, and Foxp^3^+ T-regulatory cells, without any ambiguity, and have dosimetrically superior performance over therapeutic preparations of IVIg.
4. Since the anti-HLA-E IgG2a mAbs TFL-006 and TFL-007 bind to *β*2-microglobulin-free heavy chains of HLA-I loci, it is an ideal diagnostic tool to monitor the contamination of *β*2-microglobulin-free HC of HLA-I loci in the Luminex HLA class I single-antigen beads; the presence of which can produce misleading results in monitoring transplant patients' Abs against intact HLA-I antigens (namely, *β*2-microglobulin-associated HC of HLA-I).

In view of the functional capabilities of both the anti-HLA-E IgG2a mAbs TFL-006 and TFL-007, there is a need to adapt both TFL-006 and TFL-007 for human use, in order to assess the therapeutic efficacy and potential of replacing IVIg for desensitization of organ transplant patients. Furthermore, these mAbs can be extended for other human autoimmune diseases in which IVIg is used as a therapeutic agent.

## Figures and Tables

**Table 1:** Different therapeutic preparations of IVIg show extensive HLA-Ia and HLA-Ib reactivity, which is strikingly similar to the IgG2a monoclonal Abs developed in mice by immunizing with heavy chain of HLA-E^R^ [46, 47].

Different therapeutic preparations of IVIg versus different kinds of anti-HLA-E monoclonal antibodies	Reactivity of different HLA class I antigens					
	Classical HLA-Ia alleles			Nonclassical HLA-Ib		
	A	B	Cw	E	F	G
Maximum alleles tested	31	50	16	2	1	1
Therapeutic IVIg preparations						
IVIg (GlobEx, India)	20	39	16	2	1	1
IVIg (GammaStan, USA)	31	50	16	2	1	1
IVIg (Octagam, Mexico)	30	47	16	2	1	1
IVIg (Sandaglobulin, Euro)	30	47	16	2	1	1
Anti-HLA-Ib antibodies						
TFL-006 (IgG2a)	31	50	16	2	1	1
TFL-007 (IgG2a)	26	44	16	2	1	1
TFL-037 (IgG2b)	14	39	15	2	0	0
TFL-033 (IgG1)	0	0	0	2	0	0

**Table 2:** The blastogenesis of PHA-activated CD4+ T cells was inhibited by anti-HLA-E mAbs TFL-006 and TFL-007 but not by negative control mAbs or by HLA-Ia mAbs or by nonpolyreactive HLA-E mAb TFL-037. Protein concentrations of culture supernatants are not given as they contain several exogenous and endogenous proteins. *p*^2^ refers to two-tailed *p* value (for further details see [47]).

Negative control mAbs	Mean	SD	*p* ^2^
No PHA	264	14	
With PHA	969	117	0.0005^∗^
PHA + Hu IgG1 control	1758	84	NS^∗∗^
PHA + mAb 2124 (anti-HLA-A11/A43)	1758	84	NS^∗∗^
PHA + mAb 9123 (anti-HLA-Ia+)	1435	276	NS^∗∗^
Experimental anti-HLA-E mAbs			
PHA + mAb TFL-006 (1/100) 8.870 mg/ml	502	184	0.02^∗∗^
PHA + mAb TFL-007 (1/100) 6.270 mg/ml	640	137	0.03^∗∗^
PHA + mAb TFL-037 (1/100) 6.000 mg/ml	911	54	NS^∗∗^

*p* ^2^ refers to 2-tailed *p* value. ^∗^The *p* value for “with PHA” is against “no PHA”;

^∗∗^the other *p* values are against “PHA only.”

**Table 3:** Comparison of the dose-dependent suppression of the blastogenesis of PHA activated CD4+/CD8− and CD4−/CD8+ T cells between anti-HLA-E mAb TFL-006 (supernatants (s) and purified ascites (a)) and IVIg, documenting the increased suppressive potential of TFL-mAb compared to that of an IVIg preparation. Protein concentrations of culture supernatants are not given as they contain several exogenous and endogenous proteins. *p*^2^ refers to two-tailed *p* value (for further details, see [47]).

Treatments	Blastogenesis						
	CD3+/CD4+				CD3+/CD8+		
	Concentration	Mean	SD	*p^2^*	Mean	SD	*p^2^*
TFL-006 (IgG2a) culture supernatant							
No PHA		192	14		78	11	
PHA alone		1190	91	0.002	364	59	0.01^∗^
PHA + murine IgG [1/100]		1033	92	NS	332	64	NS^∗∗^
PHA + TFL-006s [1/10]	5.000 *μ*g/ml	231	59	3*E*−04	70	25	0.007^∗∗^
PHA + TFL-006s [1/20]	2.500 *μ*g/ml	320	79	0.003	117	32	0.008^∗∗^
PHA + TFL-006s [1/40]	1.250 *μ*g/ml	575	63	0.004	204	20	0.03^∗∗^
PHA + TFL-006s [1/80]	0.625 *μ*g/ml	894	73	0.02	298	26	NS^∗∗^
PHA + TFL-006s [1/160]	0.313 *μ*g/ml	904	91	0.02	275	29	NS^∗∗^
TFL-007 (IgG2a) ascite supernatant							
No PHA		190	3		70	3	
PHA alone		1243	106	0.003	403	31	0.003^∗^
PHA + murine IgG [1/100]		1330	166	NS	422	37	NS^∗∗^
PHA + TFL-006a [1/100]	8.870 *μ*g/ml	478	193	0.008	176	75	0.02^∗∗^
PHA + TFL-006a [1/200]	4.435 *μ*g/ml	568	173	0.008	191	72	0.02^∗∗^
PHA + TFL-006a [1/400]	2.218 *μ*g/ml	588	195	0.01	207	67	0.02^∗∗^
PHA + TFL-006a [1/800]	1.109 *μ*g/ml	786	127	0.009	248	16	0.005^∗∗^
PHA + TFL-006a [1/1600]	0.555 *μ*g/ml	1499	158	NS	477	52	NS^∗∗^
IVIg octagam (6 gm%) lot A913A6431							
No PHA		46	8		53	13	
PHA alone		1685	89	<0.0001	1951	171	<0.0001^∗^
PHA + IVIg (1/10)	6.0 mg/ml	945	87	5*E*−04	1134	13	0.001^∗∗^
PHA + IVIg (1/20)	3.0 mg/ml	1365	100	0.019	1717	198	NS^∗∗^
PHA + IVIg (1/40)	1.5 mg/ml	1796	81	NS	2280	127	NS^∗∗^

*p* ^2^ refers to 2-tailed *p* value; ^∗^the *p* value for “PHA alone versus no PHA”; ^∗∗^the other *p* values are against PHA alone.

**Table 4:** Comparison of the unique features of the mAbs TFL-006 and TFL-007 with that of IVIg.

Source, nature, and functions	Intravenous immunoglobulin (IVIg)	TFL-006 and TFL-007
Manufacturer	Several pharmaceutical firms	Terasaki Foundation Laboratory
Source	Purified from pooled plasma of 10,000 blood donors from humans in various countries	Immunized in mice with heavy chain of HLA-E^R107^ [45, 46]
		Murine (to be adapted for human use), not humanized
Nature of antibody	Human, polyclonal IgG with trace level of IgA	Murine, ascites purified monoclonal IgG
Subclass of IgG antibodies	IgG1, IgG2a, IgG3, IgG4	IgG2a only [45, 46]
Purity	Contains soluble HLA antigens and other non-IgG proteins, cytokines, and chemokines	100% purified protein of IgG2a [45, 46]

Antibody reactivity	HLA-A, HLA-B, HLA-Cw, HLA-E, HLA-F, HLA-G	HLA-A, HLA-B, HLA-Cw, HLA-E, HLA-F, HLA-G [45, 46]
	HLA-DR, HLA-DQA/DQB, HLA-DPA/DPB	None
	Fc-receptors: Fc*γ*I, Fc*γ*II, Fc*γ*III, Fc*γ*IV (tested) [98]	FcgII (anticipated)
	Blood groups A, B, Rh [99–101]	Not applicable
	*Escherichia coli* bacterial antigens ranging from 94 to 238	Not applicable
	Antigens by different preparations of IVIg [102]	Not applicable
	Human albumin [46, 80]	Not applicable
	Phospholipids [100]	Not applicable
Stabilizer	Many	None
Protein concentration	Highly variable from 2 to 12%	Protein concentration can be adjusted to requirement
CD4+ T cell suppression	PHA or cytokine activated T cells [35, 36] by apoptosis [38]	PHA-activated T cells [47]
	By nonapoptosis (necrosis) [41],	
CD8+ T cell proliferation	PHA-activated [36]	PHA-activated T cells [47]
B cell proliferation	Induce differentiation [42]	
	No effect on proliferation [43]	
Anti-HLA antibody suppression	PRA antibody reduction [44]	Reduction in the production of anti-HLA-I and anti-HLA-II IgG [48]
	Induce antibody secretion [42, 48]	
	Suppress selected HLA-II antibody production [48]	Suppress production of all HLA-II antibodies [48]
	Promote selected HLA-II antibody production [48]	
Expansion of Tregs	Promotes upregulation of Tregs [39]	Promotes upregulation of Tregs [M. Taniguchi and M. H. Ravindranath, manuscript in preparation] to distinguish *β*2-microglobulin-associated HC of HLA-Ia from *β*2-microglobulin-free HC of HLA-Ia coated on Luminex single-antigen beads (One Lamda/Thermofisher Inc) [91, 92]
Special application		
	
	

## Abbreviations

Abs: Antibodies
AT1R: Angiotensin receptor 1
DSA: Donor-specific antibodies
EBV: Epstein-Barr virus
FDA: Federal Drug Administration (US)
HC: Heavy chain
HIV: Human immunodeficiency virus
HLA: Human leukocyte antigens
IL-6R: Interleukin-6 receptor
IVIg: Intravenous immunoglobulin
mAb: Monoclonal antibody
MFI: Mean fluorescent intensity
MHC: Major histocompatibility complex
NDSA: Nondonor-specific antibodies
PBMC: Peripheral blood mononuclear cells
PHA: Phytohemagglutinin
PRA: Panel reactive antibodies
TFL: Terasaki Foundation Laboratory
TRALI: Transfusion-related acute lung injury
Tregs: T-regulatory cells.
